# Phylo-Epigenetics in Phylogeny Analyses and Evolution

**DOI:** 10.3390/genes15091198

**Published:** 2024-09-12

**Authors:** Simeon Santourlidis

**Affiliations:** Epigenetics Laboratory for Human Health and Longevity, Institute of Transplantation Diagnostics and Cell Therapeutics, Medical Faculty, Heinrich-Heine University Duesseldorf, Moorenstr. 5, 40225 Duesseldorf, Germany; simeon.santourlidis@med.uni-duesseldorf.de

**Keywords:** phylo-epigenetics, transgenerational epigenetic inheritance, CpG island, CpG dinucleotides, hominids, evolution

## Abstract

Long-standing, continuous blurring and controversies in the field of phylogenetic interspecies relations, associated with insufficient explanations for dynamics and variability of speeds of evolution in mammals, hint at a crucial missing link. It has been suggested that transgenerational epigenetic inheritance and the concealed mechanisms behind play a distinct role in mammalian evolution. Here, a comprehensive sequence alignment approach in hominid species, i.e., *Homo sapiens*, *Homo neanderthalensis*, *Denisovan human*, *Pan troglodytes, Pan paniscus*, *Gorilla gorilla*, and *Pongo pygmaeus*, comprising conserved CpG islands of housekeeping genes, uncover evidence for a distinct variability of CpG dinucleotides. Applying solely these evolutionary consistent and inconsistent CpG sites in a classic phylogenetic analysis, calibrated by the divergence time point of the common chimpanzee (*P. troglodytes*) and the bonobo or pygmy chimpanzee (*P. paniscus*), a “phylo-epigenetic” tree has been generated, which precisely recapitulates branch points and branch lengths, i.e., divergence events and relations, as they have been broadly suggested in the current literature, based on comprehensive molecular phylogenomics and fossil records of many decades. It is suggested here that CpG dinucleotide changes at CpG islands are of superior importance for evolutionary developments. These changes are successfully inherited through the germ line, determining emerging methylation profiles, and they are a central component of transgenerational epigenetic inheritance. It is hidden in the DNA, what will happen on it later.

## 1. Introduction

C.H. Waddington presented conceptual explanations for how the approx. 200 different cell types, despite all having the same genetic constitution, contribute each differently, within the shortest time, to the successful development of the evidently complex human fetus, individually adapted and prepared for the upcoming environmental conditions and challenges [1]. Notably, over the last 40 years, we have gained comprehensive knowledge of the epigenetic mechanisms behind, in particular, DNA methylation, and annotating and reorganizing the genome, involved in achieving directed, meaningful cell-type-specific functionality and phenotype. Hence, epigenetics has proceeded to become of similar fundamental importance as classical genetics, and any branch of biology, e.g., evolution, and of medicine, e.g., cancer research, can only be better understood if epigenetics is appropriately taken into account.

However, the nature of the epigenetic master initiator within the fertilized oocyte, inherited by the parental generation, is an unsolved mystery. It leads to the imposition of a specific epigenetic profile, evidently and evolutionary shaped by the experiences of many ancestral generations in intensive, daily confrontations with challenging environmental conditions and requirements.

Nevertheless, currently, the neo-Darwinian view of evolution with selection as its central pillar still dominates the upcoming conceptions on transgenerational inheritance of acquired traits, which are closer to Lamarckian inheritance. The latter are controversially discussed, in particular, when they are linked to postulated transgenerational inheritance of acquired DNA methylation patterns, since primordial germ cells essentially erase DNA methylation [2].

Regardless, the complete organism, which originated in one zygote, ultimately testified to versatile evolutionary developments and adaptations, and in different models, i.e., different species. Here, what classical genetics has mostly denied, the plasticity of epigenetics and its connection to the environment provide new explanations for the requested dynamic variations in evolutionary speeds to overcome and adapt to fluctuating and challenging environmental conditions.

Currently, the ‘molecular-clock principle’ is widely used to assess the timescale of organismal evolution [3], to infer time and correlation between lineage divergence time and concurrent environmental changes [4]. Its assumption is that the number of molecular character changes strikingly reflects the phylogenetic distance of organisms and therefore allows the inference of the exact time point since the divergence of species. In addition, simplified, equal evolutionary rates are assumed between the species to be compared.

On the other hand, the scientific literature reveals yet unexplained, impressive discrepancies since molecular time scales frequently have suggested that lineage origination may be twice as old as fossils imply. For example, metazoan phyla originated several hundred million years (ca. 750–1200 Ma) before the Cambrian explosion (ca. 560 Ma) of these animals in the fossil record. And further, most orders of birds and mammals appear to have originated within the mid-late Cretaceous (ca. 80–100 Ma), while the fossil record proposes a sudden appearance of modern lineages in the early Tertiary (65–54 Ma) [4]. Interestingly, in this context, the authors conclude that the effects of the environment on shaping bird and mammal biodiversity through time are optimally testable for diversification, not origination, and they pointed out that the time of diversification within avian order and family appears to correlate with the two largest environmental changes in Tertiary history [4].

Thus, evolutionary tracking based solely on genetic variation remains difficult and blurred. Understanding the manifestation and role of epigenetic inheritance could lead to new approaches to more accurately track evolutionary processes.

The most studied epigenetic mechanism is DNA methylation, which occurs at CpG dinucleotides and is involved in cell-type-specific epigenetic suburbanization of the genome in active, silent, and transcriptional competent parts. It participates in the control of the outcome of the genetic content of a particular cell. More than 60% of human genes possess a 0.4–2 kb long, CpG-rich 5′-gene area of regulatory importance, surrounding the transcriptional start site, which is called a ‘CpG-island’. In particular, these CpG-islands of housekeeping genes remain unmethylated throughout development and in all tissues, which is thought to protect them from erosion [5]. Full and partial methylation at specific, single CpG dinucleotides within these CpG islands affect gene expression [6]. In contrast to the spontaneously occurring hydrolytic cytosine deamination, which leads to uridine in DNA and is removed by uracil-DNA glycosylase, the same reaction at methylcytosine yields the normal DNA base thymine, which largely persists at the affected positions, and this results in increased mutation rates of CpG to TpG and CpA, respectively, in both strands, due to the palindromy of the CpG dinucleotide and the methylation maintenance property of the DNA-methyltransferase 1 (DNMT-1) [7]. The transition rate of methylated CpG to TpG is 10–50 times higher than other transitional changes [8]. From this, it would generally be assumed that steadily unmethylated CpGs within CpG islands of conserved genes should also remain evolutionary conserved.

In contrast, it has been suggested that CpGs seem to be hotspots of mutations related to speciation [9]. The authors postulated that, on the one hand, certain CpG-related mutations promote genomic ‘flexibility’ in evolution, i.e., the ability of the genome to expand its functional possibilities; on the other hand, CpG-related mutations in SNPs relate to genomic ‘specificity’ in evolution, thus representing mutations that would associate with phenotypic traits relevant for speciation [9]. Furthermore, within orthologous CpG islands, those distinctive regulatory regions, associated with up to two-thirds of vertebrate gene promoters, CpG dinucleotides differ greatly in terms of base composition and frequency among vertebrate species, contributing to a faster evolution than those of CpG-poor promoters [6]. Recently, it has been suggested that even when CpGs are absent from the core transcription factor (TF) binding motif, the methylation status of CpGs influences their surrounding TF binding sites [10]. The authors conducted comprehensive epigenomic analyses on interspecies differences that arose from over 96 million years of evolution among five species and revealed coordinated evolution between transcription factor binding divergence and DNA methylation patterns. They conclude that specific DNA methylation profiles determine TF binding across species, which is associated with regulatory activity, chromatin contexts, and evolutionary trajectories [10].

Going in a similar direction, it is hypothesized here that alterations on CpG dinucleotides play a distinct evolutionary role, by direct comparison to other nucleotide changes, firstly due to their clearly increased mutation rate when methylated, and secondly, by their broadly proven functional relevance for gene expression. If so, evolutionary changes within certain CpGs, considered separately from all other nucleotide positions that undergo changes in lower rates, may reflect interspecies evolutionary relationships in a more enhanced resolution than conventional molecular analyses, and presumably, this could preferentially become visible if closer related species are analyzed [11]. Further, it is assumed, in analogy to interspecies single-nucleotide variations, that CpG dinucleotides should vary less between closely related species, which have diverged recently, as opposed to cases of earlier divergence. Thus, CpG dinucleotides have a distinct role in evolution, and may they therefore harbor and provide an improved phylo-epigenetic tool to more accurately assess phylogeny relations? When yes, how to prove it, and how to explain their role within a requested epigenetic transgenerational inheritance, since at least, at the intragenerational scale, erasure of methylation in the germ line has been proven?

To test this hypothesis, and especially to find a suitable paradigm that might demonstrate the distinct importance of CpG mutations, the following experimental approach has been developed. Two dozen conserved housekeeping genes were chosen, their CpG islands were picked up, and they were lined up one after the other. They were aligned between the selection of closely related species from which it can be assumed that they evolve at a comparable speed. All conventional non-CpG mutations were compared and evaluated versus the CpG transitions and transversions. This highly conserved scenario might reveal even slight differences in the relative frequency of the non-CpG versus the CpG mutations and, furthermore, their different significance for the manifestation of the species relationships. If such differences exist, they should penetrate here and become visible in appropriate phylogenetic tree analyses. The chosen species were primates, i.e., homo, gorilla, chimpanzee, bonobo, orang-utan, and to hopefully obtain a more reliable calibration point; in addition, the Altai Neanderthal and Denisova genomes were chosen [12].

## 2. Materials and Methods

Conserved housekeeping genes were chosen from a list provided by the Tel Aviv University server at https://www.tau.ac.il/~elieis/HKG/ (accessed on 2 October 2023) and based on the publication of Eisenberg et al. on human housekeeping genes [13]. The 5′regions of the following two dozen genes of *H. sapiens* were picked up from the nucleotide database of the National Library of Medicine at https://www.ncbi.nlm.nih.gov/ (accessed on 5 October 2023). The CpG islands of these genes were lined up one behind the other in this order: *ACTR1A*, *CALR*, *AKIRIN1*, *H3-3B*, *BTF3*, *EED*, *AKIRIN2*, *AP1B1*, *HUS1*, *ITCH*, *SIRT2*, *PUM1*, *HAT1*, *HDAC2*, *H1FX*, *TUBB*, *PCNA*, *PNN*, *POLE3*, *POMP*, *SMU1*, *TOX4*, *NCL*, and *RING1*. The homologous primate sequences of chimpanzee, bonobo, gorilla, and orang-utan derived from BLAT search from the assemblies, panTro6 (chimp), panPan3 (bonobo), gorGor6 (gorilla), ponAbe3 (orang-utan) at https://genome.ucsc.edu/cgi-bin/hgBlat (accessed on 10 October 2023) [14]. The homologous Altai Neanderthal and Denisova sequences derived from the genome browser, JBrowse 1.12.1, at https://bioinf.eva.mpg.de/jbrowse/ (accessed on 4 November 2023) [12]. All CpG-rich 5′regions were inspected by Repeatmasker at https://www.repeatmasker.org/ (accessed on 6 November 2023), to exclude repetitive DNA elements, e.g., *LINE-1*, *Alu*, etc.

**Figure 1:**
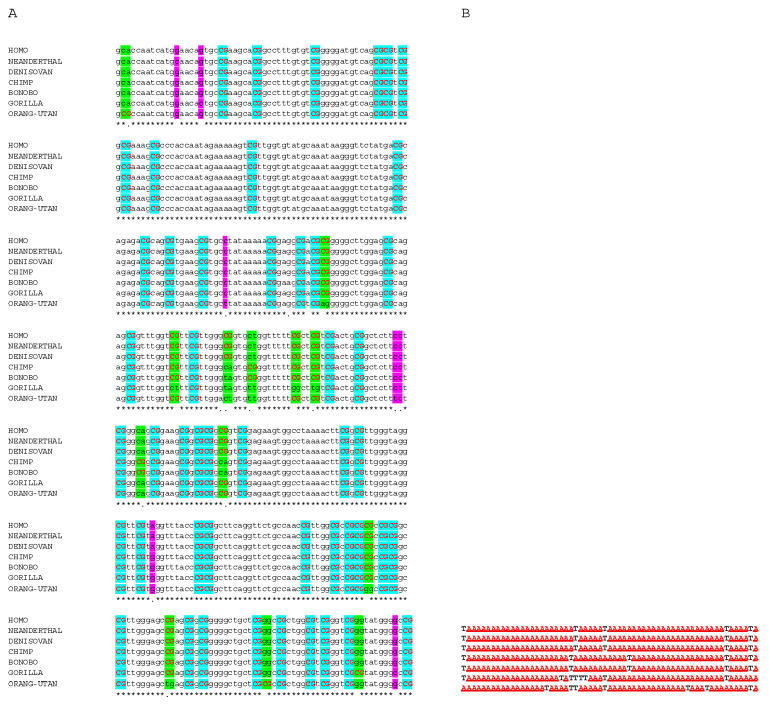
(**A**) Representative excerpt of the alignment of assembled 24 5′ CpG-rich sequences (CpG islands) of conserved housekeeping genes in hominids. All single-nucleotide polymorphisms (SNPs) outside CpGs present in at least one of the seven species are highlighted in purple (approx. 4.8% of all possible, lined-up dinucleotide positions). All CpG dinucleotides largely preserved but affected by at least one nucleotide alteration in at least one of the species are highlighted in green (that is approx. 3.2% of all possible, lined up dinucleotide positions and 17% of all CpG positions). All CpG dinucleotides consistently preserved in all species are highlighted in light blue. CpGs are highlighted in red. (**B**) Representative excerpt of the corresponding A/T alignment in which only the CpG positions are depicted; i.e., all CpGs, including those consistent in all seven species were replaced by “A”, and those altered in at least in one of the species were replaced by a “T”. *: conserved nucleotide: change of purine by purine or pyrimidine by pyrimidine.

All these sequences containing the lined-up CpG islands one behind the other were aligned by the multiple alignment program for nucleotide sequences MAFFT (v7.511) at https://mafft.cbrc.jp/alignment/server/ (accessed on 15 November 2023) using the default settings, except scoring matrix for nucleotide sequences: 1PAM/κ = 2, when aligning closely related DNA sequences and I use the iterative refinement method FFT-NS-i [15,16]. For reconstructing the phylogenetic tree, the UPGMA (unweighted pair group method with arithmetic mean) at https://mafft.cbrc.jp/alignment/server/phylogeny.html (accessed on 16 November 2023) was used, which is considered to be a simple agglomerative hierarchical clustering method that assumes a constant rate of evolution (molecular clock hypothesis) [17]. The analyses were performed after the replacement of all CpGs, which are consistent and part-consistent in all CpG-rich sequences in all seven species. Here, every CpG, consistent in all orthologous sequences, was replaced by an “A” and every same-positioned dinucleotide in every species of a position with at least one CpG in any of the other species was replaced by a “T” (Figure 1B). Visualization and comparison of the phylogenetic trees were performed by phylo.io at http://phylo.io/ (accessed on 18 November 2023) [18]. Percent Identity Matrix was created by Clustal 2.1 at https://www.ebi.ac.uk/ (accessed on 23 November 2023) [19]. 

**Figure 2:**
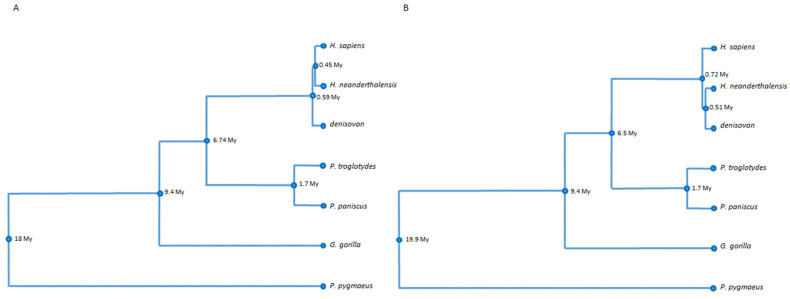
Phylogenetic (**A**) and “phylo-epigenetic” (**B**) relations between 7 primate species based on genetic and epigenetic, respectively, differences in CpG islands of 24 conserved housekeeping genes. The phylogram of the left panel displays the phylogenetic relation of these primate species, based on single nucleotide changes including CpG sites, small deletions, and nucleotides gains. The phylogram of the right panel displays the “phylo-epigenetic” relation of these species, based on all consistently occurring CpG dinucleotides and all CpG dinucleotide differences in these CpG islands of conserved housekeeping genes.

The divergence time point of the common chimpanzee (*P. troglodytes*) and the bonobo or pygmy chimpanzee (*P. paniscus*), that is, 1.7 My [20], was chosen as the calibration point for the phylogeny trees of Figure 2.

## 3. Results

An alignment of the chosen 24 CpG-rich, lined-up 5′-regulatory regions of conserved housekeeping genes from all seven species reveals the grade of conservation (an excerpt of this alignment of 420 nt is presented in Figure 1A). The sequences aligned were of a total length of 27,061 nucleotides from *H. sapiens*, 27,061 from *H. neanderthalensis*, 27,060 from Denisovan human, 27,037 from *P. troglodytes*, 27,072 from *P. paniscus*, 27,040 from *G. gorilla*, and 27,037 from *P. pygmaeus*. The sequence homologies are for example 99.9% between *H. sapiens*, *H. neanderthalensis*, and Denisovan human. The differences between these species and *P. troglodytes*, *P. paniscus*, *G. gorilla*, and *P. pygmaeus* are 99%, 99%, 98.6%, and 97.3%, respectively. All sequence homologies between these seven sequences are listed in detail in %, in Table 1. The largest difference appears between the hominins and *P. pygmaeus* with 97.18%. 

Thus, these sequences of lined up 24 CpG-rich 5′-regions (CpG islands) of conserved housekeeping genes are highly conserved among these hominid species, with sequence homologies between 99.9% and 97.2%.

All single-nucleotide positions outside CpGs, mutated at least in one of the primate sequences within the whole alignment are ca. 2.4% of the ca. 27,060 nucleotides. In order to compare the mutation rate of mutations at non-CpG dinucleotides with those at CpG dinucleotides, the ca. 27,060 nucleotides of the entire sequence were divided by 2, and all lined-up dinucleotides were again inspected for mutations. Thus, 4.8% of those ca. 13,530 dinucleotide positions were mutated at non-CpG positions at least in one of the species. In 3.2% of these, 13,530 dinucleotide positions were mutated at orthologous dinucleotide positions where a CpG is present in at least one of the species. We have in total 2495 CpG dinucleotide positions within all possible lined up 13,530 dinucleotide positions and of those 17% were altered at least in one of the species. A total of 2080 CpG dinucleotide positions were conserved throughout all species. 

By counting, the following numbers of transitions and transversions were found: they are present at dinucleotide positions that show one CpG dinucleotide in at least one of the species: CG to TG 127 (5.1%), CG to CA 109 (4.4%), CG to GG 59 (2.4%), CG to CC 45 (1.8%), CG to CT 44 (1.8%), and CG to AG 31 (1.2%). Regarding solely the CpG transitions to TpG and CpA from the perspective of every single species, Table 2 depicts that the lowest number of 94 transitions at dinucleotide positions with a TpG or a CpA at that place in at least one of the other species is present in *G. gorilla* and the highest number of 115 in *P. pygmaeus*. Of note, CpA corresponds to TpG in the complementary strand, which might result from a hydrolytic deamination of a methylated CpG. The corresponding numbers from all the other species are presented in Table 2. 

A representative excerpt of the alignment, by labeling to illustrate the relevant, described positions and their changes is shown in Figure 1A. For this part of the alignment, Figure 1B illustrates the corresponding “A/T” alignment, which refers exclusively to the throughout in all species conserved and non-conserved CpG positions (A) and to CpG dinucleotide-linked transitions and transversions (T), orthologically and equally placed with one CpG dinucleotide in at least one of the species (Figure 1B). Thus, within the sequence of conserved CpG islands of conserved housekeeping genes, a significant alteration rate persists at CpG dinucleotides and, hereby, predominantly CpG to TpG and CpA transitions are present. 

Finally, the UPGMA phylogenetic trees, one based on all single-nucleotide changes including CpG sites, small deletions, and nucleotides gains (Figure 2A) and one based solely on consistent CpG dinucleotides and CpG alterations, as explained (Figure 2B), preferably referred to here as a phylo-epigenetic tree, are presented (Figure 2). The first one displays a closer relation of *H. sapiens* to *H. neanderthalensis* than to Denisovan humans. All the other branchpoints and branches indicate divergence events and species relations, respectively, as they are established in the literature. The tree of Figure 2B resembles the phylogenetic tree of Figure 2A, with the difference that *H. neanderthalensis* appears nearer to Denisovan humans than to *H. sapiens*. When both trees are calibrated by the divergence time point of the common chimpanzee (*P. troglodytes*) and the bonobo or pygmy chimpanzee (*P. paniscus*), that is, 1.7 My [20], the phylo-epigenetic tree displays evolutionary distances between the species as has been suggested [21,22]. Thus, the resulting phylo-epigenetic tree based solely on all CpG dinucleotides and CpG dinucleotide differences reveals an image of these interspecies relations that precisely fit the current established knowledge in this field with decades of research.

## 4. Discussion

The most phylogenetically distant great ape from humans with the most ancestral karyotype among all hominids, providing an informative perspective on hominid evolution, is the orang-utan species [21]. The speciation of orang-utan is thought to have occurred no earlier than the Middle Miocene (12–16 Myr ago), as fossil apes before that differ substantially from what we might expect of an early great ape [22]. Based on comprehensive statistical analyses of molecular data and proper fossil calibration, the divergence date for gorillas and the human/chimpanzee clade have been suggested to range from 7 to 9 MYA and for humans and chimpanzees from 4 to 6 MYA. This is generally compatible with the known primate fossil record or recent molecular studies [23].

The common chimpanzee (*P. troglodytes*) and the bonobo or pygmy chimpanzee (*P. paniscus*) represent the closest living hominid species to humans and the most recently diverged ape species (around 1.7 million years ago) [20].

It has been shown that Neanderthals contributed genetically to modern humans outside Africa 47,000–65,000 years ago, and it has been proposed that the ancestors of Neanderthals from the Altai Mountains and early modern humans met and interbred many thousands of years earlier than previously thought [24]. The analysis of a Neanderthal genome from a cave in the Altai Mountains in Siberia suggests they diverged 550,000–765,000 years ago. The analysis of a Denisovan genome from the same cave in the Altai Mountains further suggests that Neanderthals and Denisovans diverged 381,000–473,000 years ago [24]. 

The main result of this study is that for the evolution of these hominid species a “phylo-epigenetic tree” has been built up, which is based solely on evolutionary consistent and inconsistent CpG sites (Figure 2B). It is noteworthy that it precisely recapitulates branch points and branch lengths, i.e., divergence events and rates of genetic changes and relations, as they have been broadly proposed and constitute the current state of knowledge in this research field, based on comprehensive molecular phylogenomics and fossil records [21,22]. This demonstrates the overriding importance of these CpG sites, which are capable of bearing the gene-regulating, epigenetic mark, methylation. 

Within this evolutionary comparison of hominids, even in highly conserved housekeeping genes with conserved sequences of CpG islands, the individual CpGs show a considerable dynamic rate of mutations, which are predominantly CpG to TpG and CpA transitions. A possible explanation might be that individual methylated CpG positions within these conserved CpG islands were preserved during the demethylation events of the early preimplantation developmental stage to become afterward subjected to spontaneous hydrolytic deamination. This occurred before the primordial germ cells of the new generation were separated. From then on, these mutations persist within the lineage, engaging the positions of former CpG dinucleotides, which presumably, originally, and decisively impacted gene expression in dependence on their methylation state. Hence, these mutations should have an altering impact on the regulation of these genes. It has been already proposed by others that a stochastic, incomplete removal of DNA methylation marks takes place during a window of opportunity in the zygote and early embryo and that genes affected are sensitive to dosage and this may be associated with evolutionary advantages [8]. Others have suggested that mutations that globally affect epigenetic marking and expression variability are potentially advantageous in a variable environment [25]. That is, in respect of methylated CpG-linked hydrolytic deamination events, opposed to the fact that a complete deletion of DNA methylation in the primordial germ cells has been demonstrated [2]. It is suggested here that this is due to methodological limitations in detecting occasional events of persisting punctual methylation, stochastically and infrequently occurring in an evolutionary timescale, or/and hydrolytic deamination has already altered methylated CpGs, before separation of the primordial germ cells, thus masking them.

The work of hundreds of laboratories has established CpG islands as distinctive regulatory regions within the vast excess of the genomic DNA sequence [6]. It is broadly accepted that methylation on certain, single CpGs influences gene expression by the spatial impediment of transcription factor binding sites for their accessibility and by influencing the DNA impact on nucleosomal positioning around the transcription start sites [6]. Therefore, it is inferred that the substitution of a cytosine by a thymine at such CpG positions of regulatory importance would impact the expression of the corresponding genes. 

Given that we are looking at conserved housekeeping genes, which prepossess a fundamental and essential role in the phenotype and function of every cell, we have to conclude that subtle evolutionary changes at these CpG dinucleotides might have a significant impact on the associated traits.

Further, it is hypothesized here that comparable analyses like this one would reveal distinct CpG profiles that act as epigenetic hotspots of evolution (EHE). It is expected that these EHEs display significantly enhanced CpG alteration rates in evolutionary time scales due to their association with genes of a basic role in the development of plastic traits, which have to flexibly adapt to meet fluctuating environmental conditions. In this respect, I am eager to see comparable studies like this on CpG islands, which are essential for dynamic embryonic development, e.g., *OCT4*, and tissue-specific expression profiles, e.g., *MyoD*. They constitute 5% of all CpG islands [6].

It is suggested here that a part of epigenetic inheritance is provided by effective turnover mechanisms, which, in an evolutionary time scale, are able to bypass early embryonic epigenetic resetting and lead to mutations at regulatory important CpG dinucleotides, which formerly had differentially impacted gene regulation dependent on their methylation state. In this scenario, it has to be requested that until now, unknown mechanisms should be able to restore, in evolutionary time scales, new CpG dinucleotides at exactly these relevant positions. To sum up, it is postulated here that evolutionary-shaped fluctuations at exactly these influential CpG sites are of superior importance for plasticity in evolution. In this context, it is accentuated that Bernhard Horsthemke and Adrian Bird refer recently [2] to the study of Takahashi et al. [25], which shows that the privileged immunity to DNA methylation of a CpG island can be seriously compromised by transient local alteration of its DNA sequence, and an acquired aberrant methylation pattern can be transmitted across generations. In their concluding remarks, they pointed out that we now need to understand the molecular mechanisms that underlie this abrupt change in epigenetic status. Importantly, the relevance of this model system to the acquisition and transmission of naturally occurring epigenetic variation has yet to be established [2]. 

It can be postulated that, on an evolutionary timeline, the selective alteration of individual CpGs in the CpG islands decisively changes the profile of these distinctive regulatory regions with regard to their immunity to methylation. This would result in significant changes in the regulation of the corresponding genes. A provocative conclusion at this point would be that precisely these changing CpGs at all genome-wide regulatory CpG positions represent an epigenetic master initiator that determines the inherited, and the environmentally shaped, genome-wide DNA methylation profile established in the early embryonic stage, henceforth determining the direction of development. It is hidden in the DNA, what will happen on it later. 

Finally, similar phylo-epigenetic analyses will be conducted, supported by an appropriate algorithm, comprising substantially larger parts of the genome, to uncover to what extent the approach presented here can further dissect phylo-epigenetic relationships in detail.

## 5. Conclusions

It is concluded that, in evolutionary time scales, changes in differentially methylatable CpG dinucleotides within CpG islands play a superior role in evolutionary developments. It is postulated that these changes are successfully inherited through the germ line, determining emerging, embryonic methylation profiles that dictate developmental fate and are a central component of transgenerational epigenetic inheritance.

## Figures and Tables

**Table 1:** Percent Identity Matrix for 24 CpG-rich 5′-regions of conserved housekeeping genes of all seven hominid species.

	Human	Neanderthal	Denisovan	Chimp	Bonobo	Gorilla	Orang-Utan
Human	100	99.90	99.89	99.01	99.01	98.63	97.32
Neanderthal	99.90	100	99.90	98.98	98.98	98.60	97.29
Denisovan	99.89	99.90	100	98.99	98.99	98.62	97.31
Chimp	99.01	98.98	98.99	100	99.74	98.56	97.23
Bonobo	99.01	98.98	98.99	99.74	100	98.57	97.23
Gorilla	98.63	98.60	98.62	98.56	98.57	100	97.18
Orang-utan	97.32	97.29	97.31	97.23	97.23	97.18	100

**Table 2:** The numbers of transitions to TpG and CpA in any of the species within the whole alignment from the perspective of every listed species with CpGs at the considered positions.

homo	98
neanderthal	101
Denisovan	99
chimp	95
bonobo	95
gorilla	94
orang-utan	115

## Data Availability

Data of this study are available from S.S. on request.
